# Intrahepatic biliary mucinous cystic neoplasms: clinicoradiological characteristics and surgical results

**DOI:** 10.1186/s12876-015-0293-3

**Published:** 2015-06-10

**Authors:** Chao-Wei Lee, Hsin-I Tsai, Yann-Sheng Lin, Tsung-Han Wu, Ming-Chin Yu, Miin-Fu Chen

**Affiliations:** 1 Department of Surgery, Chang Gung Memorial Hospital, Linkou, Taiwan; 2 Chang Gung University, Kwei-Shan Tao-Yuan, Taiwan; 3 Department Anesthesiology, Chang Gung Memorial Hospital, Linkou, Taiwan

**Keywords:** Cystadenoma, Cystadenocarcinoma, Biliary cystic neoplasms

## Abstract

**Background:**

Intrahepatic biliary mucinous cystic neoplasms are rare hepatic tumors and account for less than 5% of intrahepatic cystic lesions. Accurate preoperative diagnosis is difficult and the outcome differs among various treatment modalities.The aim of this study is to investigate the clinico-radiological characteristics of intrahepatic biliary mucinous cystic neoplasms and to establish eligible diagnostic and treatment suggestions.

**Methods:**

Nineteen patients with intrahepatic biliary cystadenomas and two patients with biliary cystadenocarcinomas were retrospectively reviewed. Their clinico-radiological variables and survival outcome were analyzed.

**Results:**

Of the 19 patients with biliary cystadenoma, 16 (84.2 %) were female. 11 (57.9 %) patients had symptoms before operation with the most common presenting symptom being abdominal pain. Among the patients with available data, serum and cystic fluid CA 19–9 levels were invariably elevated and the CA 19–9 level in the cystic fluid was significantly higher than that in the serum. Loculations (84.2 %) and septations (63.2 %) were the most common radiologic findings. For treatment, 11 (57.9 %) patients received radical resection by either enucleation or hepatic resection, while the remaining 8 (42.1 %) patients underwent only fenestration of liver cysts. Radical resection provided a significantly better clinical outcome than fenestration in terms of tumor recurrence (*p* = 0.018). The only two male patients with biliary cystadenocarcinoma received radical hepatic resection and achieved a disease-free survival of 16.5 months and 33 months, respectively.

**Conclusion:**

Intrahepatic biliary mucinous cystic neoplasms are rare and preoperative diagnosis is difficult. Internal septations and loculations on radiologic examinations should raise some suspicion of this diagnosis. Complete tumor excision is the standard treatment that may provide patients with better long term results after the operation.

## Background

Cystic lesions of the liver are being discovered more frequently nowadays owing to advances in abdominal imaging including sonography and computed tomography. They are mostly benign and asymptomatic [1]. The differential diagnoses are diverse and treatment modalities differ. Intrahepatic biliary mucinous cystic neoplasms, traditionally categorized as either biliary cystadenomas or biliary cystadenocarcinomas, have been reclassified into mucinous cystic neoplasms with low-, intermediate, high-grade intraepithelial neoplasia, or an associated invasive carcinoma [2]. These lesions account for less than 5 % of hepatic cystic lesions [3]. Since the first discovery in 1887 [4], many studies have been published in an effort to provide suggestions regarding the diagnosis and treatment for intrahepatic biliary mucinous cystic neoplasms. However, most of these articles have been case reports or small case series, rendering their suggestions less convincing. Regrettably, an accurate preoperative diagnosis for intrahepatic biliary mucinous cystic neoplasms remains difficult and controversy exists in regards to the appropriate treatment and follow-up. Therefore, the purpose of this study is to share the experience in our institute and to investigate the clinico-radiological characteristics of intrahepatic biliary mucinous cystic neoplasms. Suggestions on the subject of preoperative diagnosis and treatment strategy will also be provided.

## Materials and methods

**Fig. 1:**
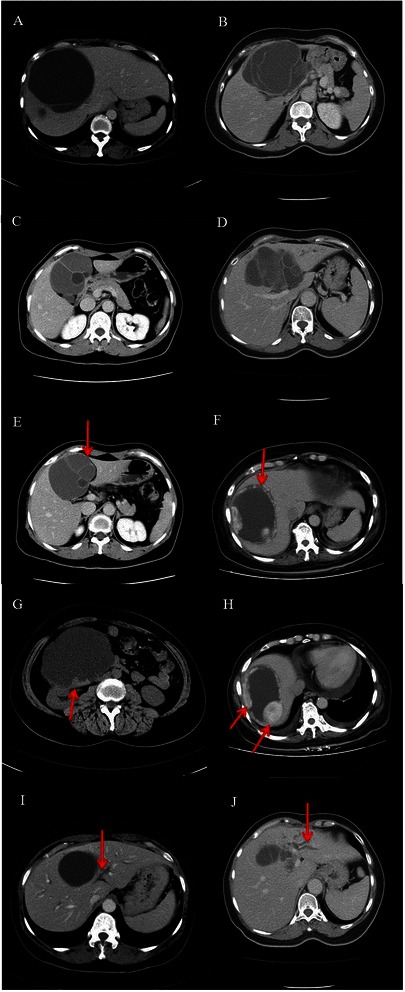
Contrast-enhanced computed tomography (CE-CT) of patients with intrahepatic biliary cystadenomas. **a** and **b**, internal septation: cystic lesion was divided by one or more septa. **c** and **d**, loculation: the presence of small spaces or cavities within the main cystic lesion. **e** and **f**, cystic wall enhancement: the enhancement of cystic wall after contrast administration. **g** and **h**, wall thickening and/or mural nodules: the presence of cystic wall nodules or focally thickened cystic wall. I and J, biliary dilatation: the presence of dilated intrahepatic ducts due to intrahepatic cystic lesion

From December 1986 to December 2012, patients with intrahepatic biliary mucinous cystic neoplasms who underwent surgery at Chang Gung Memorial Hospital, Linkou, were retrospectively reviewed. Preoperative contrast-enhanced computed tomography (CE-CT) or contrast-enhanced magnetic resonance image (MRI) of liver was routinely performed and interpreted by radiologists experienced in the hepatobiliary system. The radiologists focused their image interpretation on the presence of the following features: internal septation, loculation, cystic wall enhancement, cystic wall thickening and/or mural nodules, and biliary dilatation, as demonstrated in Fig. 1.

The patients received surgical interventions after extensive evaluations and the operations were performed exclusively by hepatobiliary surgeons in our department. The operative methods could be classified into either radical resection or liver-preserving surgery. Radical resection included tumor enucleation and anatomical hepatic resection, while liver-preserving surgery included cystic fenestration, marsupialization, and partial tumor excision. The methods of operations were left to the surgeons’ discretion. Intra-operative findings were recorded accordingly; however, no intraoperative frozen section examinations were performed.

The specimens were examined and the diagnosis of intrahepatic biliary cystadenoma and cystadenocarcinoma was made by histopathologists expertised in the hepatobiliary system. Postoperatively, the patients had regular follow-up visits every 3 months for two consecutive years and then every 6 months thereafter. Serum tumor markers and imaging studies such as ultrasonography, CT, or MRI were conducted periodically during follow-up visits. Postoperative symptom resolution, tumor recurrence, additional interventions, and survival outcome were recorded.

The statistical analysis was performed with SPSS 17.0 for Windows (SPSS Inc., Chicago, IL, USA). Fisher’s exact test or Pearson’s *χ*2 test was used to analyze categorical data. Student’s *t* test was used to analyze continuous variables. Disease-free survival (DFS) was calculated from the date of operation to the date of first documented radiological recurrence. Overall survival (OS) was defined by the time elapsing from the date of diagnosis to either the date of death or the date of the last contact. Statistical significance was defined as *p* values < 0.05.

## Results

During the study period, a total of 21 patients with intrahepatic biliary mucinous cystic neoplasms who underwent surgery at our hospital were included and examined. Among them, nineteen patients had biliary cystadenoma and two patients had biliary cystadenocarcinoma. Their respective clinico-radio-pathological findings and surgical results are listed below:

### Cystadenoma

The mean age upon diagnosis was 57 and 16 patients (84.2 %) were female. Female patients tended to be younger than male patients upon diagnosis. (54.7 vs. 69.3 year-old, *p* = 0.017). The demographic data and preoperative serum/cystic fluid tumor marker levels are shown in Table 1.

**Table 1:** The demographic data of patients with intrahepatic biliary cystadenoma (n = 19)

Variables	(%)	Variables	(%)
**Age(yr)**		**ALT(U/L)**	
≦60	12 (63.2)	≦36	3 (15.8)
>60	7 (36.8)	>36	7 (84.2)
**Gender**		**Child-Pugh Classification**	
Male	3 (15.8)	A	17 (89.5)
Female	16 (84.2)	B	2 (10.5)
**Cigarette Smoking**		**Serum AFP (ng/mL)** ^a^	
Yes	3 (15.8)	>15	0 (0)
No	16 (84.2)	Mean ± SD	3.78 ± 1.37
**Alcohol**		**Serum CEA (ng/mL)** ^a^	
Yes	3 (15.8)	>5	1 (10.0)
No	16 (84.2)	Mean ± SD	19.03 ± 49.87
**HBV**		**Serum CA 19–9 (U/mL)** ^a^	
Positive	3 (15.8)	>37	6 (100.0)
Negative	16 (84.2)	Mean ± SD	444.81 ± 480.45
**HCV**		**Serum CA 125 (U/mL)** ^a^	
Positive	0 (0)	>35	2 (50.0)
Negative	19 (100)	Mean ± SD	516.73 ± 947.25
**Total bilirubin(mg/dl)**		**Cystic CEA (ng/mL)** ^a,b^	108.07 ± 139.47
≦1.3	18 (94.7)	**Cystic CA 19–9 (U/mL)** ^a,b^	1033140.54 ± 1376297.74
>1.3	1 (5.3)	**Cystic CA 125 (U/mL)** ^a,b^	1051.26 ± 1269.11

^a^ only patients with available data were analyzed

^b^ expressed as mean ± SD

As for clinical presentation, 11 patients (57.9 %) were symptomatic, with a duration of these symptoms ranging from 10 days to 2 years. Of these, 54.5 % approached for hospital care within 1 month of onset of the symptoms. The frequency of presenting symptoms are summarized in Table 2.

**Table 2:** The presenting symptoms, radiologic findings, and surgical indications of patients with intrahepatic biliary cystadenoma

Presenting symptoms	(%)	Radiologic findings	(%)
Asymptomatic	8 (42.1)	Septation	9 (56.3)
Symptomatic	11(57.9)	Loculation	14 (87.5)
Abdominal pain	8 (72.7)	Wall enhancement	4 (25.0)
Abdominal mass	1 (9.1)	Wall thickening / mural nodules	4 (25.0)
Abdominal distension	1 (9.1)	Biliary dilatation	4 (25.0)
Fever	2 (18.2)	**Surgical indications**	(%)
Jaundice	1 (9.1)	Symptomatic relief	7 (36.8)
Dyspnea	1 (9.1)	Increased size of lesion	2 (10.6)
Diarrhea	1 (9.1)	Suspected neoplasm on radiologic examination	10 (52.6)

Radiologic examinations revealed that 14 patients (87.5 %) had loculations, demonstrating such being the most common radiologic finding among the patients with intrahepatic biliary cystadenoma. The second most common finding was internal septations (56.3 %). Cystic wall enhancement, cystic wall thickening / mural nodules, and biliary dilatation were also common features. Every patient had at least one characteristic finding mentioned above. The imaging studies of three patients were inaccessible due to prolonged history and were therefore not included in our analysis.

As for preoperative diagnosis, only 6 patients (31.58 %) received preoperative percutaneous aspiration cytology or biopsy, of which showed negative results for malignancy without specific diagnosis. The definitive diagnosis still depended on the pathological examination of excised specimen (Table 3). All 19 patients received surgical intervention for their respective intrahepatic biliary cystadenoma. The mean tumor size was 8.5 cm in diameter, with the size ranging from 2.5 cm to 21.0 cm at the largest dimension. The radiologic findings and surgical indications are summarized in Table 2.

**Table 3:** Summary of preoperative cytological and postoperative pathological diagnosis

Case No./Age/Gender	Preoperative FNAC^a^ findings	Preoperative FNAC^a^ diagnosis	Postoperative pathological diagnosis
No.2/39/Female	N.A.	Negative for malignancy	Biliary cystadenoma
No.9/49/Female	Fibrous tissue with scattered smooth muscle bundles^b^	Negative for malignancy^b^	Biliary cystadenoma with leiomyomatous mural nodules
No.10/65/Female	Columnar/cuboidal cells with eccentric nuclei	Atypia	Mucinous cystadenoma
No.11/32/Female	Lymphocytes and histiocytes	Negative for malignancy	Mucinous cystadenoma
No.12/49/Female	Lymphocytes and histiocytes	Negative for malignancy	Mucinous cystadenoma
No.19/69/Male	Lymphocytes	Negative for malignancy	Mucinous cystadenoma
No.20/73/Male^c^	Cystic spaces lined by mucinous cuboidal or columnar epithelium with papillary infoldings^b^	Cystic mucinous neoplasm^b^	Biliary cystadenocarcinoma
No.21/62/Male^c^	Complex glandular structures floating in mucin pools^b^	Mucinous carcinoma or mucinous cystadenocarcinoma or IPMN^d b^	Biliary cystadenocarcinoma

^a^fine needle aspiration cytology

^b^core needle biopsy

^c^biliary cystadenocarcinoma

^d^intraductal papillary mucinous neoplasm

**Fig. 2:**
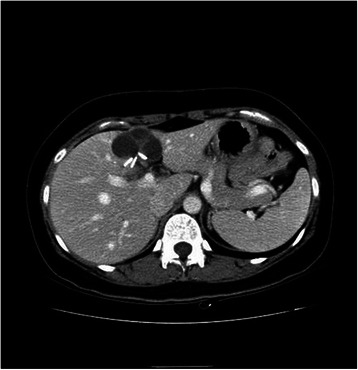
Postoperative recurrence after liver-preserving surgery. A 38 year-old female received laparoscopic cystic fenestration for intrahepatic biliary cystadenoma. Four months after the operation, tumor recurrence was documented by CT. The high density objects beside the cystic tumor were hemoclips used to secure blood vessels

As for operative methods, eleven patients (57.9 %) received radical resections, and 8 patients (42.1 %) received liver-preserving surgery. During their regular postoperative follow-up visits, no tumor recurrence occurred in patient who received radical resections; however, 5 out of 8 patients who received liver-preserving surgery(62.5 %) had tumor recurrence (*p* = 0.005). The median follow-up time in radical resection group was 51.3 months and the median disease-free survival (DFS) in liver-preserving surgery group was 3.7 months. The other 3 patients in the group of liver-preserving surgery were lost in follow-up. Among patients with postoperative tumor recurrence, 3 patients (60 %) received further surgical interventions, namely liver resections in 2 and cystic fenestration in 1. The first patient who received liver resection remained disease- and symptom-free at last follow-up. The second patient who received liver resection during her second operation had positive resection margins on the specimen and suffered from another recurrence at resection margin two months after the second operation. The only patient who had re-fenestration for his recurrence was agonized by persistent recurrent disease and right upper quadrant abdominal pain after the second operation. Of the other two patients with postoperative tumor recurrence, one developed recurrence 4.17 months after the operation and remained symptomatic throughout the follow-up, and the other had persistent disease and died from massive upper gastrointestinal bleeding 32 days after the operation. Fig 2 demonstrates postoperative recurrence after laparoscopic cystic fenestration. Table 4 summarizes the operative methods and surgical outcome of patients with intrahepatic biliary cystadenoma.

**Table 4:** The operative method and surgical outcome of patients with intrahepatic biliary cystadenoma

Operative method	(%)	Post-operative tumor recurrence	(%)	*P*-value
Radical resection*	11 (57.9)	Radical resection*	0 (0)	0.005
Liver-preserving surgery**	8 (42.1)	Liver-preserving surgery**	5 (62.5)	
		**Tumor location**		(%)
		Right lobe		5 (29.4)
		Left lobe		10 (58.8)
		Bilateral lobes		2 (11.8)

*Radical resection included enucleation and anatomical hepatic resection

**Liver-preserving surgery included cystic fenestration, marsupialization, and partial tumor excision

### Cystadenocarcinoma

The first patient with intrahepatic biliary cystadenocarcinoma was a 73 year-old male who presented with epigastric pain for weeks on a background history of chronic hepatitis B carrier. Physical and laboratory examinations were unremarkable. His serum CA 19–9 level was 111.9 U/mL. The preoperative contrast-enhanced CT showed a loculated, infiltrative and septated cystic mass with solid component and wall enhancement at segment 4 and 5. Malignancy was suspected and percutaneous biopsy showed cystic mucinous neoplasm. Segmental hepatectomy (S4 and S5), cholecystectomy and partial omentectomy were performed to remove the tumor en bloc. The final pathology reported an 8.5 cm intrahepatic biliary cystadenocarcinoma with a sclerotic stroma. Postoperative recovery was uneventful. Nevertheless, the patient developed intrahepatic recurrence and pulmonary metastasis 16.5 months postoperatively and obtained an overall survival (OS) of only 23.2 months.

**Fig. 3:**
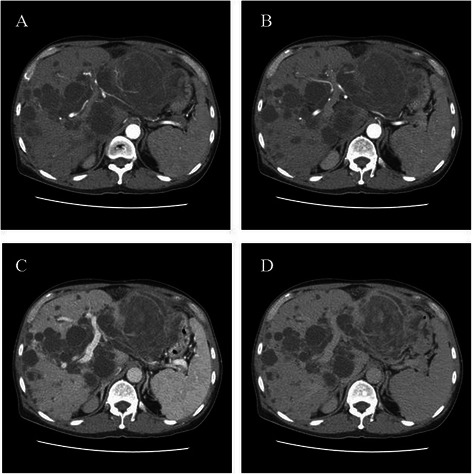
Contrast-enhanced triphasic liver CT of patient with intrahepatic biliary cystadenocarcinoma. The large (14 × 9.5 cm) cystic tumor occupying left lateral liver sector had diffuse mural soft tissue and fine tumor vessels. Faint arterial blushes with whirling appearance were also noted within the tumor and they faded throughout the dynamic study. Multiple liver cysts of various sizes were also found at the right liver lobe. **a** and **b**: arterial phase; **c**: portal venous phase; **d**: delayed venous phase

The second patient was a 62 year-old male who presented with a two-week history of abdominal pain, left upper quadrant in location and dull in character, in addition to abdominal distension on a background history ofautosomal dominant polycystic kidney and liver disease. He was also an HBV carrier. Physical examination revealed hepatosplenomegaly and left upper abdominal tenderness. His serum tumor markers were normal. Contrast-enhanced dynamic CT showed, in addition to multiple various-sized liver cysts, a large (14 × 9.5 cm) cystic mass at left lateral sector of liver. The cystic tumor had diffuse mural soft tissue and fine tumor vessels (Fig. 3). Preoperative biopsy included mucinous carcinoma, mucinous cystadenocarcinoma, and IPMN into differentials. Gastric and diaphragmatic invasion was noted intraoperatively and left lateral segmentectomy, wedge excision of gastric tumor, and partial diaphragm excision was performed to obtain an R0 resection. Pathologic interpretation reported a 16 cm intrahepatic biliary cystadenocarcinoma with metastatic carcinoma in the gastric wall. Recovery was smooth and no evidence of recurrence was found during follow-up examinations. He was still well at last follow-up visit 33 months postoperatively.

Although only two malignant cases were reported herein, statistical analysis was still conducted to compare the major clinical features between biliary cystadenoma and cystadenocarcinoma. In contrast to biliary cystadenoma, biliary cystadenocarcinoma had a male predominance and occurred at an older age (Table 5).

**Table 5:** Comparison of clinical characteristics between biliary cystadenoma and biliary cystadenocarcinoma

Variables	Cystadenoma	Cystadenocarcinoma	*P* value
**Gender**			
Male	3 (15.8 %)	2 (100 %)	0.008
Female	16 (84.2 %)	0 (0 %)	
**Age (yr)**			
≦60	12 (63.2)	0 (0 %)	0.086
>60	7 (36.8)	2 (100 %)	
**Size (cm)**			
≦5.0	5 (33.3 %)	0 (0 %)	0.331
>5.0	10 (66.7 %)	2 (100 %)	
**Serum CEA (ng/mL)** ^a^			
Mean ± SE^b^	19.03 ± 16.62	2.25 ± 0.85	0.544
**Serum CA 19–9 (U/mL)** ^a^			
Mean ± SE^b^	444.81 ± 196.14	72.61 ± 39.29	0.699

^a^only patients with available data were analyzed

^b^standard error of mean

## Discussion

The differential diagnoses of hepatic cystic lesions include simple cysts, parasitic cysts, mucinous cystic neoplasms, congenital cystic dilatation, degenerated metastatic tumors, mucin producing metastatic tumors, cystic hemangioma, lymphangioma, hepatic foregut cyst, and mesenchymal hamartoma and teratoma [5–7]. Mucinous cystic neoplasms including intrahepatic biliary cystadenoma and cystadenocarcinoma, account for approximately 5 % of hepatic cystic lesions [3]. They are being discovered more frequently due to advances in abdominal imaging modalities and improved awareness of this disease entity. Although benign, intrahepatic biliary cystadenomas have the potential for malignant transformation and the prognosis of intrahepatic biliary cystadenocarcinomas is poor [8, 9].

The World Health Organization (WHO) in 2010 had re-categorized biliary cystadenoma into mucinous cystic neoplasms with low-, intermediate, or high-grade intraepithelial neoplasia and biliary cystadenocarcinoma into mucinous cystic neoplasms with an associated invasive carcinoma [2]. However, most authors in the recent literature continue using cystadenoma/cystadenocarcinoma to describe this rare disease. For the convenience of further literature review, we adopt the terms cystadenoma/cystadenocarcinoma in the current study. Readers should be aware that these terms should be avoided in the future.

The cause of intrahepatic biliary cystadenoma remains unknown. The almost exclusive female predominance of biliary cystadenoma (>85 %) suggests a strong hormonal influence [10]. Our study, in which 84.2 % of the biliary cystadenoma patients are female, corresponds to this published result. The mean age of diagnosis in our study is 57, which is similar to most of other series [11, 12]. This feminine hormonal influence might explain the older age on diagnosis in male population (69.3 year-old in male and 54.7 year-old in female patients, respectively), of whom masculine hormone decreases with aging.

Intrahepatic biliary cystadenomas are rare. Few large series have been reported in the literature [1, 6, 12–18]. Our study is by far one of the largest series in the English literature. The clinical presentations of intrahepatic biliary cystadenomas could vary widely, as evidenced from the literature [1, 12, 16, 17]. In contrast to the previous series, in which most patients were symptomatic before operation, only 11 patients (57.9 %) were symptomatic upon presentation in the current study and 8 patients (42.1 %) were found incidentally. The most common presenting symptom was abdominal pain whereas fever, abdominal mass/distension, jaundice, dyspnea and diarrhea were the less common symptoms. The nonspecific nature of these symptoms rendered clinical diagnosis difficult and laboratory/radiologic examinations imperative. Numerous efforts were taken in the past to develop a sensitive and specific diagnostic modality for intrahepatic biliary cystadenoma. Koffron A. et al. suggested that cystic fluid analysis could be used to differentiate intrahepatic biliary cystadenomas from simple cysts, of which cystic fluid CEA and CA19-9 were usually normal [16]. Intrahepatic biliary cystadenomas, on the other hand, usually had elevated cystic fluid CEA and CA 19–9 levels. However, another study indicated that cystic fluid CEA and CA 19–9 levels were not useful in differentiating biliary cystadenoma from simple cysts [12]. They reported that both hepatic simple cysts and biliary cystadenoma could have elevated cystic fluid CEA and CA 19–9 levels. Our study did not compare the difference between those hepatic simple cysts and intrahepatic biliary cystadenomas. Nevertheless, among patients with available data, we found that serum and cystic fluid CA 19–9 levels were invariably elevated in the intrahepatic biliary cystadenomas. In addition, cystic fluid CA 19–9 level was significantly higher than that in serum. These findings could be explained by part the biliary origin of intrahepatic cystadenomas. As a result, we suggest preoperative serum and cystic fluid CA 19–9 determination to exclude intrahepatic biliary cystadenomas.

Since there are neither sensitive nor specific clinical or laboratory findings suggestive of intrahepatic biliary cystadenomas, preoperative imaging studies are thus indispensable for the evaluation of patients with hepatic cystic mass. In the current study, every patient received either contrast-enhanced CT or MRI for preoperative evaluation. Among other features, loculations and internal septations were the two most common radiologic characteristics in patients with intrahepatic biliary cystadenoma, with 87 % and 56 % of patients having these features, respectively. Our finding was in accordance with previous reports, in which septations and/or septal thickening were the common radiologic features in intrahepatic biliary cystadenomas [1, 12]. Therefore, preoperative contrast-enhanced radiologic study, including CT or MRI, should be carried out and intrahepatic biliary cystadenomas should be highly suspected if loculations and/or internal septations are demonstrated. It is noteworthy, however, that these radiologic features are not specific for intrahepatic biliary cystadenomas and the definitive diagnosis should be based on final pathologic finding.

Preoperative pathologic diagnosis remains difficult for intrahepatic biliary cystadenomas. In our series, 6 patients received preoperative percutaneous aspiration cytology or biopsy. The interpretation did not give any specific diagnosis. The reason for these false negative results may be attributed to inadequate tissue sampling upon percutaneous biopsy/aspiration since intrahepatic biliary cystadenomas usually have rather thin walls, as demonstrated by our study in which only 25 % of biliary cystadenomas had thickened wall/mural nodules. As a result, we do not suggest routine preoperative aspiration/biopsy only if the cystic neoplasm has apparent wall thickening or mural nodules and the biopsy is carried out by experienced hands.

The treatment of intrahepatic biliary cystadenomas remains controversial among literatures. Most authors suggested complete surgical removal by either liver resection or enucleation to prevent recurrence or potential malignant transformation [1, 5, 6, 11, 15, 17, 19]. Nevertheless, there have been other authors indicating that marsupialization of the hepatic cysts could result in optimal outcome without tumor recurrence [16]. In our study, none of the 11 patients who received radical resection developed recurrence postoperatively, while more than 60 % of patients who underwent liver-preserving surgery developed postoperative recurrence. Given the possibility of malignant transformation, we suggest radical resection by either hepatic resection or enucleation for suspected intrahepatic biliary cystadenomas. The emergence of laparoscopic techniques in recent decades renders the surgeons more options regarding hepatic surgery and should also be considered. Since most patients with intrahepatic biliary cystadenomas are non-cirrhotic in the absence of viral hepatitis, hepatic resection should be relatively safe, provided that adequate future liver remnant is reserved. On the other hand, in the cases of hepatic viral infection and/or cirrhosis, liver functions, particularly indocyanine green 15 min retention test (ICG-15), should be taken into consideration before performing radical hepatic resection. The result after well-planned radical hepatic resection is usually good and patients can achieve satisfactory long-term outcome.

As for intrahepatic biliary cystadenocarcinoma, a recent study has shown that older age, male gender, and shorter symptom duration were the major differences from biliary cystadenoma [18]. The current study, although with limited cases, agreed to that publication. In addition, a septated cystic mass with a solid mural component on image study should also raise our suspicion of this diagnosis [9]. As for treatment, we suggest radical resection with negative resection margin for suspected intrahepatic biliary cystadenocarcinomas. This approach has been the only potentially curative treatment proposed by most other authors [1, 17]. Reported survival rates for intrahepatic biliary cystadenocarcinomas ranged from 25 % to 100 % at 5 years [1]. Despite its poor prognosis, our patients survived for at least 2 years after the operations.

## Conclusion

Intrahepatic biliary mucinous cystic neoplasms are rare and preoperative diagnosis is difficult. High serum and cystic fluid CA 19–9 levels, together with loculated and septated cystic lesions on radiologic exams, should raise clinical suspicion of biliary cystadenoma and cystadenocarcinoma. Biliary cystadenomas occur predominantly, but not exclusively, in females. Preoperative tissue diagnosis, either by percutaneous aspiration or biopsy, may be falsely negative and thus is not routinely recommended. Percutaneous aspiration, surgical fenestration, and marsupialization are inadequate treatments and invariably result in persistence or recurrence of disease. The standard treatment of choice is complete tumor excision, either by tumor enucleation or hepatic resection. Patients can achieve satisfactory long-term results once biliary cystic neoplasm is completely resected.

## Consent

This study was approved by Institutional Review Boards of Chang Gung Memorial Hospital, and informed consent was obtained from the patient for the publication of this report and any accompanying images (IRB number: 102-5742B).

## References

[1] Vogt DP, Henderson JM, Chmielewski E (2005). Cystadenoma and cystadenocarcinoma of the liver: a single center experience. J Am Coll Surg.

[2] Bosman FT, Carneiro F, Hruban RH, Theise ND (2010). WHO Classification of Tumours of the Digestive System, Fourth Edition.

[3] Walt AJ (1977). Cysts and benign tumors of the liver. Surg Clin North Am.

[4] Henson SW, Gray HK, Dockerty MB (1957). Benign tumors of the liver. VI. Multilocular cystadenomas. Surg Gynecol Obstet.

[5] Hansman MF, Ryan JA, Holmes JH, Hogan S, Lee FT, Kramer D (2001). Management and long-term follow-up of hepatic cysts. Am J Surg.

[6] Thomas KT, Welch D, Trueblood A, Sulur P, Wise P, Gorden DL (2005). Effective treatment of biliary cystadenoma. Ann Surg.

[7] Del Poggio P, Buonocore M (2008). Cystic tumors of the liver: a practical approach. World J Gastroenterol.

[8] Woods GL (1981). Biliary cystadenocarcinoma: Case report of hepatic malignancy originating in benign cystadenoma. Cancer.

[9] Matsuoka Y, Hayashi K, Yano M (1997). Case report: malignant transformation of biliary cystadenoma with mesenchymal stroma: documentation by CT. Clin Radiol.

[10] Kim HH, Hur YH, Koh YS, Cho CK, Kim JW (2011). Intrahepatic biliary cystadenoma: Is there really an almost exclusively female predominance?. World J Gastroentero.

[11] Oh TH, Kim MH, Lee SK, Seo DW, Lee SS, Kim EY (2006). Thirteen cases of intrahepatic biliary cystadenoma and cystadenocarcinoma: a single center experience. Korean J Gastroenterol.

[12] Choi HK, Lee JK, Lee KH, Lee KT, Rhee JC, Kim KH (2010). Differential diagnosis for intrahepatic biliary cystadenoma and hepatic simple cyst: significance of cystic fluid analysis and radiologic findings. J Clin Gastroenterol.

[13] Ishak KG, Willis GW, Cummins SD, Bullock AA (1977). Biliary cystadenoma and cystadenocarcinoma: report of 14 cases and review of the literature. Cancer.

[14] Wheeler DA, Edmondson HA (1985). Cystadenoma with mesenchymal stroma (CMS) in the liver and bile ducts. A clinicopathologic study of 17 cases, 4 with malignant change. Cancer.

[15] Lewis WD, Jenkins RL, Rossi RL, Munson L, ReMine SG, Cady B (1988). Surgical treatment of biliary cystadenoma. A report of 15 cases. Arch Surg.

[16] Koffron A, Rao S, Ferrario M, Abecassis M (2004). Intrahepatic biliary cystadenoma: role of cyst fluid analysis and surgical management in the laparoscopic era. Surgery.

[17] Emre A, Serin KR, Ozden I, Tekant Y, Bilge O, Alper A (2011). Intrahepatic biliary cystic neoplasms: Surgical results of 9 patients and literature review. World J Gastroenterol.

[18] Wang C, Miao R, Liu H, Du X, Liu L, Lu X (2012). Intrahepatic biliary cystadenoma and cystadenocarcinoma: an experience of 30 cases. Digestive Liver Dis.

[19] Lee JH, Lee KG, Park HK, Lee KS (2009). Biliary cystadenoma and cystadenocarcinoma of the liver: 10 cases of a single center experience. Hepatogastroenterology.

